# Chitosan Functionalized Magnetic Nanoparticles to Provide Neural Regeneration and Recovery after Experimental Model Induced Peripheral Nerve Injury

**DOI:** 10.3390/biom11050676

**Published:** 2021-04-30

**Authors:** Nadina Liana Pop, Alexandrina Nan, Andrada Elena Urda-Cimpean, Adrian Florea, Vlad Alexandru Toma, Remus Moldovan, Nicoleta Decea, Daniela Rodica Mitrea, Remus Orasan

**Affiliations:** 1 Department of Physiology, Iuliu Hațieganu University of Medicine and Pharmacy Cluj-Napoca, Clinicilor Street No. 1-3, 400006 Cluj-Napoca, Cluj County, Romania; popnadina@yahoo.com (N.L.P.); remus_ri@yahoo.com (R.M.); nicoleta_decea@yahoo.com (N.D.); rorasan@yahoo.com (R.O.); 2 National Institute for Research and Development of Isotopic and Molecular Technologies, Donath Street No. 67-103, 400293 Cluj-Napoca, Cluj County, Romania; alex77nan@gmail.com; 3 Department of Informatics and Biostatistics, Iuliu Hațieganu University of Medicine and Pharmacy Cluj-Napoca, Pasteur Street No. 4-6, 400349 Cluj-Napoca, Cluj County, Romania; aurda@umfcluj.ro; 4 Department of Cell and Molecular Biology, Iuliu Haţieganu University of Medicine and Pharmacy, Pasteur Street No. 4-6, 400349 Cluj-Napoca, Cluj County, Romania; aflorea@umfcluj.ro; 5 Department of Molecular Biology and Biotechnologies, Babeș-Bolyai University, Clinicilor Street No. 4-6, 400000 Cluj-Napoca, Cluj County, Romania; vlad.al.toma@gmail.com; 6 Institute of Biological Research, Republicii Street No. 48, 400015 Cluj-Napoca, Cluj County, Romania

**Keywords:** functional rehabilitation, peripheral nerve injury, chitosan magnetic nanoparticles, sciatic functional index

## Abstract

(1) Background: Peripheral nerve injuries have a great impact on a patient’s quality of life and a generally poor outcome regarding functional recovery. Lately, studies have focused on different types of nanoparticles and various natural substances for the treatment of peripheral nerve injuries. This is the case of chitosan, a natural compound from the crustaceans’ exoskeleton. The present study proposes to combine chitosan benefic properties to the nanoparticles’ ability to transport different substances to specific locations and evaluate the effects of magnetic nanoparticles functionalized with chitosan (CMNPs) on peripheral nerve injuries’ rehabilitation by using an in vivo experimental model. (2) Methods: CMNPs treatment was administrated daily, orally, for 21 days to rats subjected to right sciatic nerve lesion and compared to the control group (no treatment) by analyzing the sciatic functional index, pain level, body weight, serum nerve growth factor levels and histology, TEM and EDX analysis at different times during the study. (3) Results: Animals treated with CMNPs had a statistically significant functional outcome compared to the control group regarding: sciatic functional index, pain-like behavior, total body weight, which were confirmed by the histological and TEM images. (4) Conclusions: The results of the study suggest that CMNPs appear to be a promising treatment method for peripheral nerve injuries.

## 1. Introduction

Peripheral nerve injuries represent a medical challenge for physicians regarding the finding of an ideal treatment for each patient: non- or minimally invasive, capable of enhancing proper nerve regeneration, with minimal or no future disability and disturbance of the patient’s life quality.

It is estimated that in Europe alone, there are more than 300,000 upper extremity injuries per year, which results in an urgent need to accelerate peripheral nerve regeneration techniques and technologies [1].

The present golden-standard treatment for peripheral nerve injuries is surgery with the development of peripheral nerve microsurgery in the 1960s, and since then, no other specific treatment has been recognized as proper for peripheral nerve injuries. In many cases though, surgery is not possible due to the patient’s disapproval, the nature and localization of the nerve lesion, or too much time lapsed from the nerve injury occurrence [2]. In these cases, a conservative treatment is initiated, which mainly includes a combination between medication and a rehabilitation program. Unfortunately, in motor function impairment, the rehabilitation outcomes remain delayed, unpredictable, and usually incomplete [1]. Until now, the medication currently used is mostly symptomatic and cannot offer satisfactory or long-term symptom amelioration in all cases. Therefore, finding a treatment aiming peripheral nerve regeneration and also pain relief, which would provide a reduced rehabilitation period of time represents a necessity.

Pathophysiologically, when a peripheral nerve injury develops, first demyelination of axons by Schwann cells (SCs) occurs [3], which have the ability to shift to a non-myelinating regenerative state and continue to deliver neurotrophic factors and attract macrophages to the injury site [4,5]. In some peripheral nerve injuries, the blood-nerve barrier is compromised, which permits macrophages to migrate to the injury site, where they produce factors supporting Schwann cell proliferation and phagocytosis of debris [5]. In these cases, a minimal proximal demyelination or degeneration is observed and proximal neurons are directed to a regenerative state [5,6]. Wallerian degeneration quickly occurs in the distal segment, and macrophages and Schwann cell phagocytosis clear the myelin debris to promote axonal regeneration [4,6]. Next, Schwann cells proliferate and restore the extracellular matrix to build pathways for axons to regenerate. Schwann cells continue to sustain regeneration by secreting pro-regenerative growth factors (GFs) and incorporating neural cell adhesion molecules in the endoneurial connective tissue [7], like nerve growth factor (NGF) and fibroblast growth factors, which preserve the regenerating microenvironment for axonal elongation after nerve injury [8]. NGF is part of the neurotrophins family, brain signaling molecules of the essence for synapse maturation and plasticity, axon targeting and neuron growth [9]. In vivo studies demonstrated that NGF stimulates axonal regeneration, electrophysiological, and histomorphological parameters following nerve injury [10]. 

The interest of finding different treatment methods for peripheral nerve injuries has increased recently and researchers studied various combinations of nanoparticles with several agents in order to improve known therapies or to find new ones. One study combined iron-oxide magnetic nanoparticles (MNPs) with 100 ng of NGF, which revealed that they did not promote any inflammatory processes and did not interfere with the physiological regeneration process after median nerve induced lesion in rats [11].

Recently, medical research has centered around various natural compounds in combination with nanomolecules. Chitosan is such a natural compound, obtained by alkaline deacetylation of chitin, the major component of crustaceans’ exoskeleton. Chitosan is biocompatible, hydrophilic, non-toxic, and has a good chemical and thermic stability [12,13]. Studies revealed that chitosan has specific physical and chemical properties that simulate peripheral nerves’ structure when it is part of artificial nerve grafts. A 2019 review discusses the regenerative axonal potential of chitosan and its capacity to enhance functional rehabilitation and to reduce the frequency of post-surgery neurinoma [14]. Studies also showed that chitosan does not cause inflammatory response and presents low toxicity when administrated orally [15]. These particular properties make chitosan a viable oral treatment option for peripheral nerve injuries and also for other types of pathologies. 

Bendable chitosan nerve guides used in gap repair demonstrated a good recovery of thenar muscle reinnervation, skilled forelimb reaching, and positive electrodiagnostic findings (in vivo model), representing a good alternative treatment for small nerve defects in a mobile extremity region [16]. Chitosan nerve tubes stimulate Schwann cells and axonal regeneration by enhancing the number of SCs and by having a good neuroglial cell affinity and low cell toxicity for SC [14]. Moreover, in 2014, a chitosan-based nerve conduit, Reaxon^®^ Nerve Guide manufactured by Medovent GmbH (Mainz, Germany), was launched for human use, for the treatment of small dimensions’ nerve lesions (not exceeding 26 mm) [17]. 

Several in vitro studies presented some properties of chitosan. It may be the substrate for Schwann cell survival and can orientate their growth, [18] and it can enhance the neuronal cells survival and differentiation [19,20]. Literature data revealed that animals treated with chitosan tubes surgical repair, at an early regeneration phase (21 days’ analysis after nerve injury) presented a higher number of activated Schwann cells in the distal segments of nerves. The authors considered that, in this way, Schwann cells are stimulated and attract the outgrowing axons [21].

On the other hand, magnetic nanoparticles (MNPs) are being used as drug delivery systems since the 1970s, when Zimmermann and Pilwat [22] utilized magnetic erythrocytes for the delivery of cytotoxic drugs and Widder et al. described magnetic albumin microspheres encapsulated with doxorubicin in animal models [23].

Magnetic nanoparticles are utilized as drug delivery systems for their ability to target specific locations in the body, to reduce the needed quantity of drug and have minimum side effects by reducing the concentration of the drug at non-target sites. Nanoparticles (NPs) are considered efficient drug delivery systems with effects at the cellular level, where they can be internalized by endocytosis or phagocytosis, so they are able to pass even the nuclear membrane in some situations [24]. 

Magnetic nanoparticles have a large specific surface area and superparamagnetism. Among magnetic nanoparticles, the most frequently used are iron oxide nanoparticles, like magnetite (Fe_3_O_4_) nanoparticles due to their superior magnetic properties [25].

Moreover, iron oxide nanoparticles (IONPs) have gained a wide research interest in the last years referable to their intrinsic magnetic properties, biocompatibility, stability, and eco-friendliness [26]. Plus, IONPs were approved by the Food and Drug Administration (FDA) as contrast agents for magnetic resonance imaging (MRI), suggesting that IONPs are safe for human use [27]. Studies showed that drug loading or bounding onto iron oxide nanocarriers can improve these drugs’ therapeutic effects due to the magnetic and biological properties of the IONPs, and by conjugation, IONPs are able to eliminate unfitted properties of most of these drugs, such as poor solubility, high toxicity, nonspecific delivery, and short circulating half-lives [27]. 

IONPs can target specific sites by passive or active targeting. Passive targeting is achieved by enhanced permeability and retention (EPR) effect, which was mostly studied in oncology [28]. Active targeting refers to guiding IONPs to specific locations using an external magnetic field (magnetic targeting) or by functionalizing their surface with vectors able to interact with given biomarkers [28]. 

The special properties of IONPs make them attractive magnetic drug delivery systems, currently being the most popular in drug delivery systems in recent studies [29].

The elimination of nanoparticles from systemic circulation by reticuloendothelial system (RES) depends mostly on the particle size. Larger superparamagnetic iron oxide nanoparticles (SPIONs) depend on passive targeting and are quickly taken by the reticuloendothelial system in Kupffer liver cells, and have limited uptake in lymph and bone tissues. On the other hand, smaller SPIONs (under 50 nm) present slower opsonization and clearance from the reticuloendothelial system, circulate for longer and are gradually taken up by the reticuloendothelial system in lymph tissue and bone marrow [30] and have the ability to remain for a long period of time (up to seven weeks) in slowly dividing human mesenchymal stem cells [31]. The uptake mechanism of the nanoparticles into biological systems may differ, according to the types of cells or to the nanomaterials used in the experiments. Two endocytic pathways, phagocytosis and pinocytosis, have been identified as possible transport mechanisms for nanoparticles. Pinocytosis is involved in cellular uptake of fluids and particles with sizes <0.5 µm, whereas large particles (>0.5 µm) are taken by phagocytosis, which implicates macrophages, dendritic cells and neutrophils [32].

Different studies regarding IONPs toxicity reported that they induce oxidative stress through reactive oxygen species (ROS) generation and mitochondrial dysfunction by the Fenton mechanism [32]. The IONPs toxicity was dose and time dependent, and was mostly against the hepatocytes and caused by modifications of the antioxidant enzyme levels, generating DNA damage and apoptosis [32]. Literature data revealed that chitosan, part of chitosan functionalized magnetic nanoparticles—CMNPs, has the ability to protect and stabilize the magnetic nanoparticle, which makes it non-detectible for the immune system and useful for future functionalization [33]. Moreover, magnetic nanoparticles functionalized with chitosan had a relatively reduced cytotoxicity [27,34]. Studies disclosed that chitosan in combination with magnetic nanoparticles are efficient drug carriers for breast cancer cells, and, by encapsulating the drug particles on the surface of the nanoparticles, it can control the drug release at the specific site [35]. To the best of our knowledge, CMNPs have not been studied as a conservative treatment method for peripheral nerve injuries up until present. The objective of this study is to bring the medical research in the domain of peripheral nerve injuries’ treatment a step forward in finding a compound capable of producing nerve regeneration, pain relief, safe, with minimal side effects. On the other hand, the latest research focused on studying iron oxide nanoparticles, especially in the field of cancer therapy, and we considered that expansion of the subject area would provide a wider knowledge of promising future medical applications. Moreover, we hypothesized that the combination of IONPs and chitosan will enhance the advantages of both and that the slow elimination of the IONPs could provide a medium- to long-term conservative treatment method option for peripheral nerve injuries. Our purpose was to assess the major symptoms of a peripheral nerve injury that are present in humans (loss of function of the affected limb, motor impairment, persistent pain, hyperesthesia, hyperalgesia, or allodynia) and to observe if CMNPs are able to provide any kind of improvement. In consideration of this purpose, we analyzed different parameters, as described below and we used an experimental design that permitted histological and ultrastructural analysis and provided detailed information about the effects of CMNPs on a peripheral nerve injury and even adverse reactions analyzed in future studies. 

## 2. Materials and Methods

### 2.1. Preparation and Characterization of Magnetic Nanoparticles Covered by Chitosan

The magnetic nanoparticles (MNPs) were prepared by the well-known co-precipitation method from FeCl_2_ and FeCl_3_ with a molar ratio of 1 to 2 in the presence of aqueous ammonia, procedures previously reported in the literature [36,37]. All the other reagents used in this study were purchased from Sigma Aldrich (St. Louis, MO, USA) and Alfa Aesar by ThermoFisher Scientific (Kandel, Germany) and did not require further purification.

**Scheme 1:**
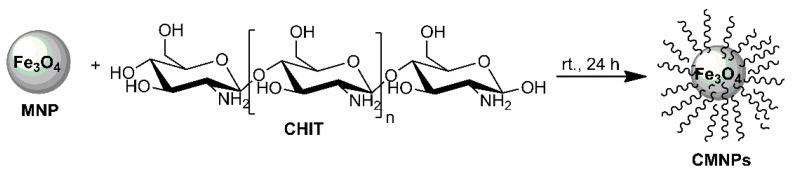
Coating of magnetic nanoparticles with chitosan (MNP—magnetic nanoparticles, CHIT—chitosan, CMNPs—chitosan magnetic nanoparticles, rt—room temperature).

The resulting MNPs were coated with chitosan (CHIT) (Scheme 1), MNPs (1 g) were dispersed in distilled water (50 mL) and then chitosan (2 g) was added and the suspension was allowed at room temperature for 24 h. After the achievement of the adsorption process of chitosan on the MNPs surface, the chitosan functionalized magnetic nanoparticles (CMNPs) were magnetically separated by an external magnet and washed consecutively with water and methanol, respectively, in order to remove all free chitosan. 

Infrared absorption spectra in the 400 to 4000 cm^−1^ spectral range were recorded with a JASCO FTIR-6100 spectrophotometer (JASCO GmbH, Pfungstadt, Deutschland) as a pressed pellet prepared from the MNPs powder embedded in KBr (potassium bromide). The morphology of the MNPs was characterized by transmission electron microscopy (TEM) using a Hitachi HD-2700 scanning transmission electron microscope (Hitachi High-Tech, Krefeld, Germany). Magnetic measurements were performed at room temperature using a vibrating sample magnetometer manufactured by Cryogenics (Cryogenic Limited, London, UK).

Coating of magnetic nanoparticles by chitosan was confirmed by FTIR spectroscopy. For comparison, FTIR-spectra of MNP as well as of CMNPs were included. 

### 2.2. Experimental Design

Sixteen white Wistar male rats, weighted between 100 and 260 g, age between 16 and 20 weeks (provided by the “Iuliu Hatieganu” University of Medicine and Pharmacy’s Research Base, Cluj-Napoca, Romania) were used. The animals were randomly distributed into two groups: eight rats for the control group—peripheral nerve injury (right sciatic nerve) without treatment and eight rats for the experiment group—peripheral nerve injury (right sciatic nerve) treated with CMNPs.

The study observed the ethical principles regarding animal research: the principle of the 3Rs (reduce, refine, replace). In concern to the present ethical guidelines, a minimal number of animals was used, just above the limit that allows a proper statistical processing and analysis. The animals were closely supervised and during the experiment, none of the animals presented any signs, symptoms, or diseases that would have imposed the withdrawal from the study (significant distress or pain, lesion site infection, other diseases, death). The present study was approved by the University Ethical Board and the Veterinary and Food Safety Direction (project authorization no. 204/10.03.2020). 

For peripheral nerve injury, an approximately 2.5 cm skin incision was made at right femoral eminence for all animals under intraperitoneal anesthesia (ketamine 40 mg/kg and xylazin 8 mg/kg). An approximately 3 mm segment of the right sciatic nerve was compressed and strangulated at 1–1.2 cm proximal to nerve’s trifurcation, with a non-resorbable 5.0 nylon surgical wire for 15 seconds. After that, the surgical wire was loosened and remained attached to the nerve in order to mark the nerve defect and to facilitate the histological studies. All surgical procedures and animals’ monitoring were performed by the same two persons. For the sciatic nerve, a standardized evaluation method for the functional nerve regeneration was used [38].

The experimental group received a solution of magnetic nanoparticles functionalized with chitosan, starting the next day after the peripheral nerve injury, daily, for 21 days. Each animal received a 2.5 mg/kg/day of CMNPs, in a NaCl 0.9% sterile solution (1 mL of solution containing 0.0145 g of CMNPs), administrated by gavage, without anesthesia. The dosage was established according to the literature data that used chitosan for different treatment methods or for nanotoxicity evaluation [39,40]. The control group received the same dosage of a simple NaCl 0.9% solution. 

### 2.3. Monitoring of CMNPs Treatment Efficacy 

#### 2.3.1. Sciatic Functional Index Assessment

The treatment efficiency was appreciated by sciatic functional index (SFI) calculated before peripheral nerve lesion induction (T0), and after the nerve damage in three moments: day 7 (T1), day 14 (T2), and day 21 (T3). 

SFI calculation is a standardized method to evaluate the functional rehabilitation level after an experimental peripheral nerve lesion and represents the method used in all in vivo studies involving a sciatic nerve injury since it was described by Medinaceli et al. and modified by Bain et al. [38]. 

SFI measurement regards the mark left by the animal’s foot, in a method similar to the one developed by Medinaceli et al. and modified by Bain et al.: the two posterior feet of the animal are impregnated with blue ink and the animal is released to move in a controlled environment (glass tunnel). Comparative measurements are done between the normal foot (N) and the experiment foot with the peripheral nerve lesion (E) and the SFI is calculated using the following formula modified by Bain: SFI = −38.3 × PLF+ 109.5 × TSF+ 13.3 × ITF −8.83 [41]. The parameters measured are: TS (toe spread), which is the distance between fingers 1–5, ITS (intermediary toe spread), the distance between fingers 2–4, PL (print length), the plantar print length. After that, the following factors are calculated: PLF (print length factor) = (EPL-NPL)/NPL; TSF (toe spread factor) = (ETS-NTS)/NTS and ITF (intermediary toe factor) = (EIT-NIT)/NIT.

The functional rehabilitation level achieved by each animal was assessed using the formula elaborated by Medinaceli et al. [38] (Table 1).

#### 2.3.2. Pain-Like Behavior Assessment

For rodents, pain cannot be directly measured but it can be assessed from “pain-like” behaviors, for example the withdrawal of the rodent’s tail or foot from a nociceptive stimulus. Such a method is analgesiometry or the Randall–Selitto test, which is the most commonly used method to quantify nociception in animal studies. The test was developed in 1957 as an evaluation method for pain levels in rodents, by measuring the animal’s resistance to mechanical pain and it is considered to be the most objective pain-like behavior assessment method by comparison with cold/heat sensibility evaluation [42]. The testing involves a mechanical force of increasing intensity applied on the animal’s foot or tail, which is maintained until the animal shows a retracement behavior (the animal retracts its foot or tail as a sign of pain) [43].

The pain-like behavior of the animals from the study was appraised on days 7, 14, and 21 using a bench-top Ugo Basile (Gemonio, Italy) analgesia-meter. 

During testing, the animals were immobilized in a cotton towel in order to ensure uninterrupted access at the animal’s foot. Previously, before the peripheral nerve injury induction, the animals were accommodated to the testing procedure and the analgesia-meter for seven consecutive days. During pain evaluation, the mechanical pressure was directed on the dorsal part of both posterior feet (normal foot—N and experiment foot—E), in the same point for each animal (between the tip of the cone-shape and the plane surface of the foot). The mechanical pressure was constantly increased until the detection of a nociceptive response. The animals’ nociceptive behavior was observed in a subjective manner, by the researcher. The maximal force applied was 300 g to prevent tissue damage or other similar problems.

#### 2.3.3. Histopathology Evaluation 

For the evaluation of histological features, the sciatic nerves were isolated and fixed in 10% neutral formalin solution for 48 h. After paraffin embedding, sections were cut at 7 μm (microtom Reichert, Austria) and mounted on glass slides. Tissue sections were dewaxed in xylene, rehydrated, and methylene blue staining was used for the sciatic nerve histological exam (Merck, Darmstadt, Germany). The sciatic nerve samples were analyzed with an incorporated camera optical microscope (Optika 383-LD2, Ponteranica, Italy). 

### 2.4. TEM and EDX (Energy-Dispersive X-ray Spectroscopy) Nerve Studies

The nerve samples collected from each group were processed for TEM according to the chemical fixation and embedding protocol (Hayat, 2000) [44]. Briefly, they were prefixed for 2 h in glutaraldehyde 2.7% in phosphate buffer 0.1 M, pH 7.4 and postfixed for 1.5 h in OsO_4_ 1.5% in phosphate buffer 0.15 M, pH 7.4. Then, the samples were dehydrated in ethanol solution of increasing concentrations (30 minutes each), and infiltrated with EMBED 812. The sections double contrasted for 10 minutes with 13% uranyl acetate and for 5 min with 2.8% lead citrate were examined on a Jeol JEM 1011 transmission electron microscope (Jeol, Tokyo, Japan), equipped with a Mega View G2 camera (Olympus Soft Imaging Solutions, Münster, Germany).

Nerve samples from the CMNPs treatment group were analyzed using a Hitachi HD-2700 scanning transmission electron microscope (Hitachi High-Tech, Krefeld, Germany), which was equipped with an X-Max 1160 energy-dispersive X-ray spectroscopy (EDX) detector (Oxford Instruments, Wiesbaden, Germany) to characterize their compositional structure. The EDX is considered a valuable instrument in every research that necessitates elemental determination, either endogenous or exogenous, in the tissue, cell, or other new types of analyses.

### 2.5. Body Weight Assessment

The animals’ body weight is another tool used in studies for the assessment of neuropathic pain or distress in rodents [45,46]. In the study, all animals were weighted before the peripheral nerve injury occurrence (T0) and at the end of the experiment, day 21 (T3). The body weight changes can be a useful tool that can indicate the animal’s general state and level of comfort, even regarding pain-like behavior. An animal that presents a moderate to intense distress will manifest a poor general state with low appetite and, weight loss. In opposition, an animal that has a low level of pain and a good general state will have a tendency to maintain a proper appetite and will have a constant weight or even a weight gain. The measurements were statistically analyzed.

### 2.6. Serum NGF Levels Analysis

Blood samples were taken from all animals of both groups prior to the sciatic nerve lesion (T0), at seven days after the lesion (T1) and at 21 days after nerve injury (T3). The blood samples were analyzed for the NGF levels detection (ELISA kit, Sigma Aldrich, Darmstadt, Germany), using a Sunrise microplate ELISA reader (Tecan, Grödig, Austria) and an Asys microplate ELISA washer (Atlantis, Austria).

### 2.7. Data Analysis

Quantitative data were described using the mean value, standard deviation, median, and the inter quartile range (Q1–Q3, the range between the 25th percentile and the 75th percentile). The Friedman test was used to determine whether there were any statistically significant differences between the distributions of the four groups for not normally distributed data. Pairwise comparisons (Wilcoxon signed-rank tests) were performed with a Bonferroni correction to compare the measurements from week 0 with each of the other weeks. The Mann–Whitney U test was applied to check if there was a significant difference between the CMNPs treatment and control groups for the non-normally distributed variables. Comparison of normally distributed data between two groups was done using a *t*test for paired samples, *t*-test for independent samples, and a Levene test for variances. A *p*-value equal to or lower than 0.05 was considered statistically significant. Data were analyzed using IBM SPSS software, v25 (manufactured by IBM). The obtained NGF values were statistically analyzed using GraphPad Prism version 5.03 for Windows, GraphPad Software, (San Diego, CA, USA), two-way ANOVA followed by the Bonferroni post-tests. The threshold significance level was set at *p* < 0.05.

## 3. Results

### 3.1. CMNPs Characterization

The strong absorption band around 580 cm^−1^ ascribing the Fe-O bond present in the magnetite molecules appeared in both the MNP FTIR spectrum and also in the CMNPs FTIR spectrum. The spectrum of CMNPs showed significant changes of peaks positions and intensities of the vibration bands as compared with MNP spectrum. The peak bands located at 2928 cm^−1^ and 2855 cm^−1^ were attributed to stretch -C-H alkane bond. The peak at 1627 cm^−1^ was attributed to the bending vibration bond of -N-H. The broad band with the peak at 1376 cm^−1^ was assigned to -C-N stretching bond and -C-H bending vibration bond. The broad large vibration band located at 1072 cm^−1^ was ascribed -C-O stretching vibration bond.

**Figure 1:**
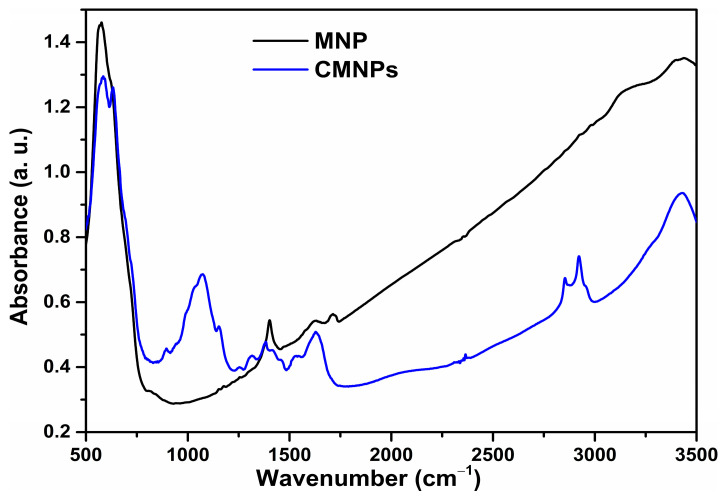
FTIR spectrum of uncovered MNP (black) and MNP covered with chitosan CMNPs (blue).

The FTIR-spectra of MNP was compared to the FTIR-spectra of the CMNPs (Figure 1). 

**Figure 2:**
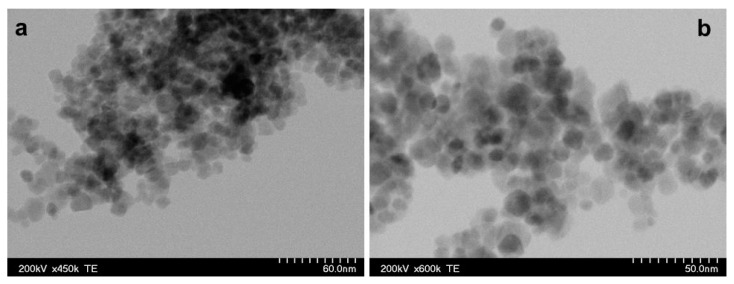
TEM images of (**a**) uncovered MNP and (**b**) chitosan functionalized magnetic nanoparticles CMNPs.

The TEM images of uncovered MNPs (Figure 2a) and the chitosan functionalized magnetic nanoparticles CMNPs (Figure 2b) documented the formation of the functionalized magnetic nanostructure. The shapes of the uncovered MNPs were nearly spherical with diameters between 9 and 12 nm; while for CMNPs, the shape was nearly the same, but the diameters were between 29 and 32 nm, that is, the diameter increased by about 20 nm as a result of the chitosan adsorption at the magnetic nanoparticles surface in a thick layer. These diameters of the magnetite core were well below the critical domain size of magnetite and, therefore, a superparamagnetic behavior was expected for the functionalized magnetic nanoparticles CMNPs [47].

**Figure 3:**
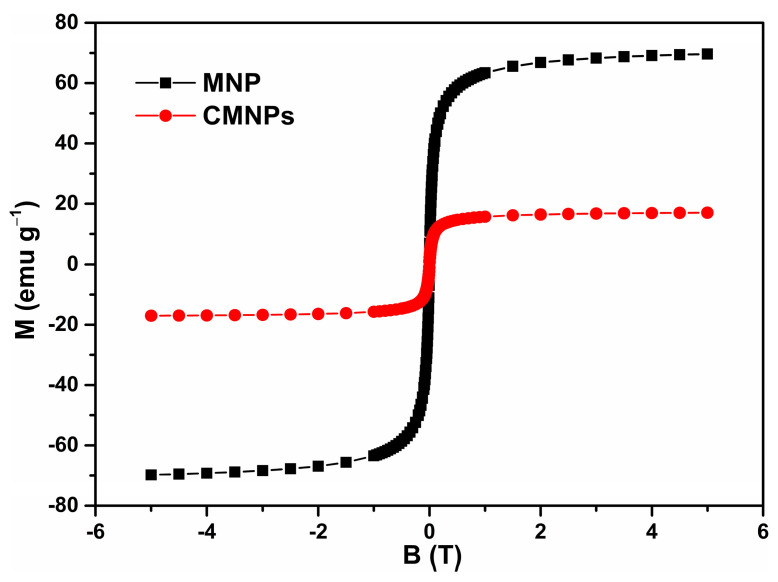
Magnetization curve of uncovered MNPs (black) and of CMNPs (red); M-magnetization saturation; B(T)-magnetic field measured at room temperature.

The magnetic properties of the uncoated MNPs and chitosan functionalized MNPs (CMNPs) were investigated by magnetometry confirming superparamagnetic behavior in both cases. The saturation magnetization of CMNPs (17 emu/g) was much lower than that of the uncoated MNPs (70 emu/g) (Figure 3) because additional mass was introduced by the chitosan adsorption at the MNPs surface. Regardless of all these differences, both magnetic nanoparticles uncoated and coated exhibited saturation magnetizations high enough for the envisaged practical application in biomedicine.

### 3.2. CMNPs Treatment Efficiency by Evaluating the SFI Score

When assessing the SFI score at different time intervals, we observed that the animals from the control group presented, as more time passed from the nerve injury occurrence, visible signs of motor impairment like foot dragging and walking difficulties compared to little or no evident impairment for the animals that received CMNPs.

**Figure 4:**
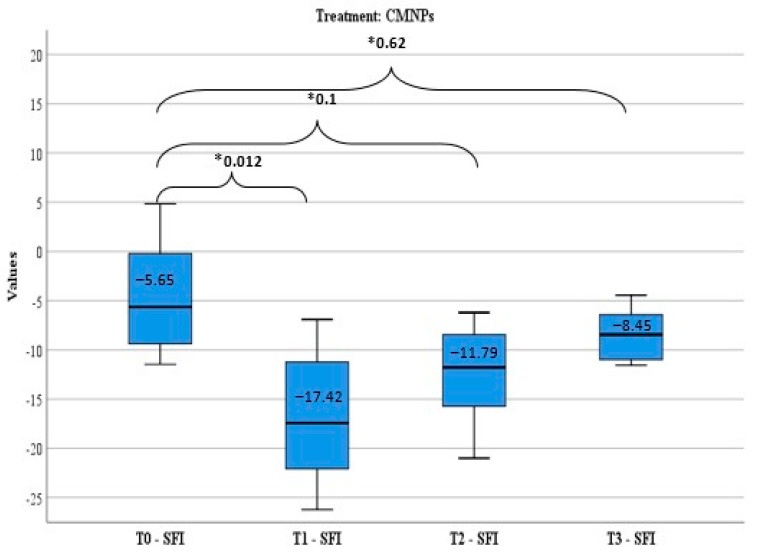
SFI score distribution (median) and evolution in time (* Wilcoxon signed-rank tests: *p*-values) for the CMNPs treatment group.

A Friedman test was run to determine if there were differences in SFI scores between T0, T1, T2, and T3. SFI was statistically significant at the different time points for the CMNPs group, χ^2^(3) = 7.86, *p* = 0.049 < 0.05. Though this *p*-value was close to non-significance, pairwise comparisons were performed with a Bonferroni correction to compare the measurements from week 0 with each of the other weeks, the significance level was set at 0.0167. The SFI median score at T0 was −5.65 (within the limits for excellent functionality), at T1 median decreased to −17.42 after the intervention, next it increased at T2 and T3 the median SFI score was close to the one observed at the beginning of the study, but the differences were not statistically significant (Figure 4).

The CMNPs group also showed a visible favorable evolution of the 25th percentile—for T0 the SFI score was −9.89; T1 presented a mild functional deterioration at seven days after peripheral nerve injury, with an ameliorating tendency at the end of the experiment. The T3 and T0 values for 25th percentile for SFI were very close. Similar tendencies were observed regarding the 75th percentile. Almost identical values were observed for T0 and T3 regarding the minimum SFI values (−11.46, respectively −11.57) (Figure 4).

**Figure 5:**
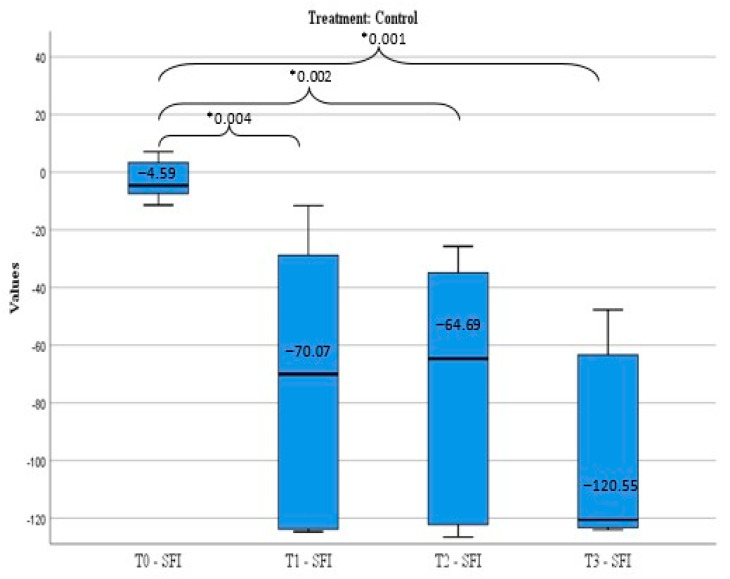
SFI score distribution (median) and evolution in time (* Wilcoxon signed-rank tests: *p*-values) for the control treatment group.

A Friedman test was run to determine if there were differences in SFI scores between T0, T1, T2, and T3. SFI was statistically significantly different at the different time points for the Control group, χ^2^(3) = 14.55, *p* = 0.002 < 0.05. Pairwise comparisons were performed with a Bonferroni correction to compare the measurements from week 0 with each of the other weeks; the significance level was set at 0.0167. Post hoc analysis revealed statistically significant differences in SFI median scores at T0 (−4.59) compared to T1 (−70.07), T2 (−64.69), and T3 (−120.55), respectively (Figure 5).

**Figure 6:**
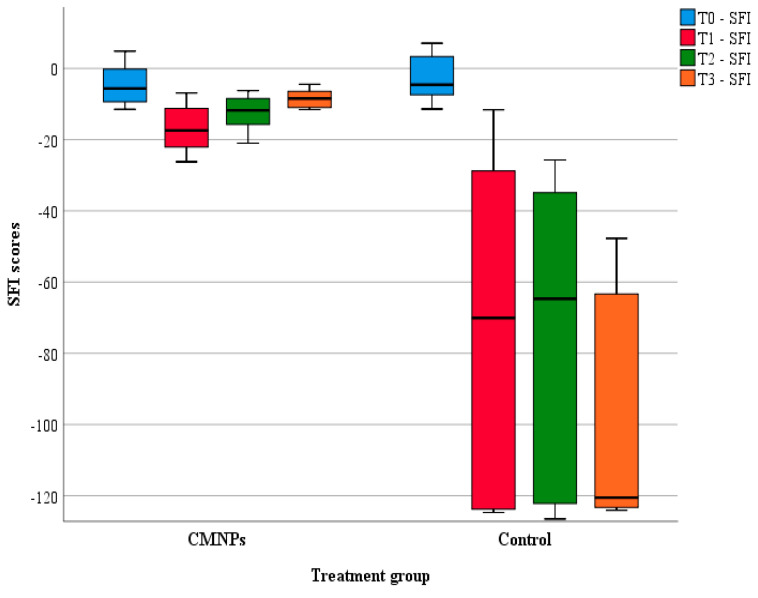
Comparative analysis of the SFI score between control group and CMNPs treatment group (dynamic evaluation).

For the control group, there was a significant reduction from T0 to T1 for the 25th and 75th percentiles, and minimum value. From T1 to T3 we observed a constant decreasing tendency. We observed that T0 minimal SFI value of −11.39 decreased to −124.07 at T3, while T0 maximal values 7.07 decreased up to −47.72 at T3. The treatment evaluation continued with the comparison between the SFI scores of the two groups for each moment in time (Figure 6).

A Mann–Whitney U test was run to determine if there were differences in SFI scores between CMNPs and control groups. Distributions of the SFI scores for CMNPs and control groups were not similar, as assessed by visual inspection (Figure 6). There was no statistically significant difference in T0 SFI scores’ distribution between CMNPs and control groups, U = 23, z = −0.9451, *p* = 0.34 > 0.05. This showed that the two groups were comparable in the beginning of the study.

For T1, the SFI scores for CMNPs group (mean rank = 11.63) were statistically significantly higher than for the control group (mean rank = 5.38), U = 27, z = −2.6255, *p* = 0.009 < 0.05. For T2, the SFI scores for CMNPs group (mean rank = 12.5) were statistically significantly higher than for the control group (mean rank = 4.5), U = 0.00, z = −3.3606, *p* = 0.001 < 0.05. For T3, the SFI scores for CMNPs group (mean rank = 12.5) were statistically significantly higher than for the control group (mean rank = 4.5), U = 0.00, z = −3.3606, *p* = 0.001 < 0.05.

### 3.3. CMNPs Treatment Efficiency Related to Pain-Like Behavior Assessment

**Figure 7:**
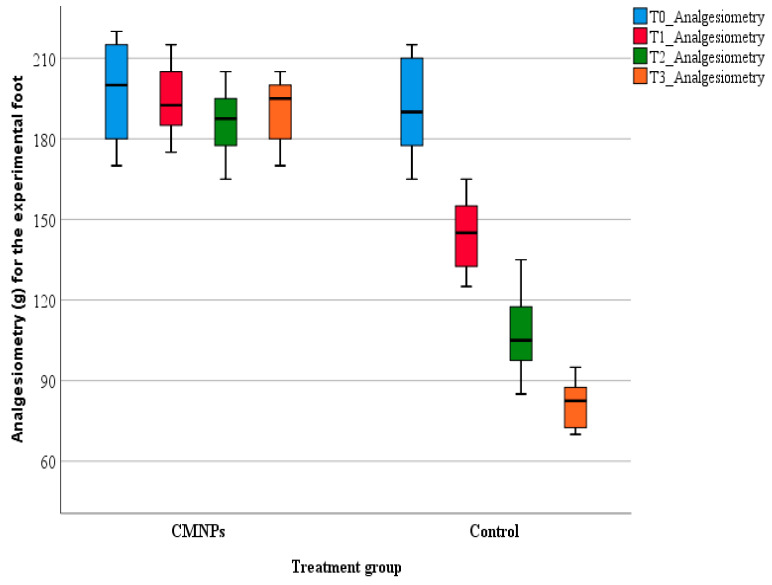
Comparative analysis of pain level between CMNPs group and control group for the experimental foot.

**Figure 8:**
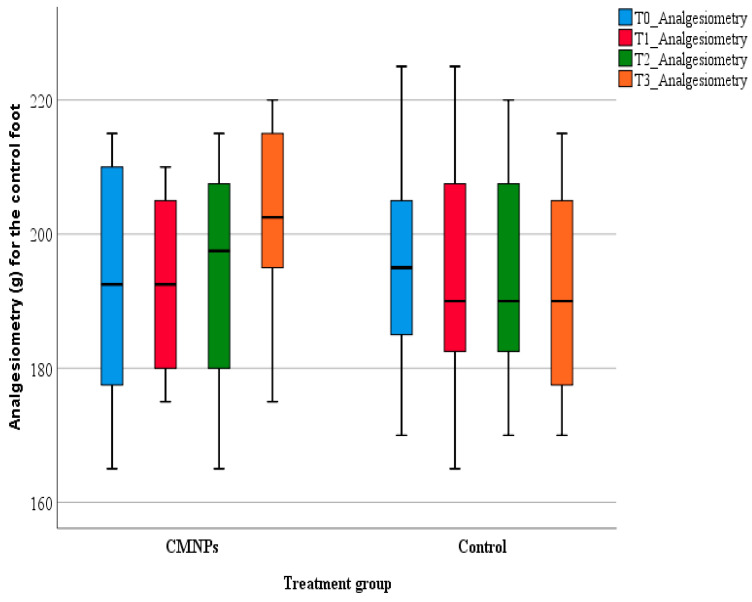
Comparative analysis of pain level between CMNPs group and control group for the normal foot.

The pain endurance was assessed for each animal from both groups by using Randall–Shapiro test, as described in the Methods section. The mechanical force (expressed in grams) that each animal could stand until the point a nociceptive response occurred was noted for the normal foot and the experimental foot before the peripheral nerve injury inducement (T0) and after the injury at 7 days (T1), 14 days (T2), and 21 days (T3). Statistical analysis was elaborated and compared the results of CMNPs group to the control group and compared the mean pain levels for the experimental foot in the two groups (Figure 7 and Figure 8).

Comparing the pain scores for T0 of the control foot (healthy foot), there were no significant differences between CMNPs group mean pain scores and control group mean pain scores for T0, T1, T2, and T3, respectively (*t*-tests for independent samples: *p* > 0.05).

Comparing the pain scores for T0 of the experimental foot, the CMNPs mean (197.5) was not statistically significant compared to the control group mean (191.88), t = 0.587, *p* = 0.567 > 0.05. Comparing the pain scores for T1, T2, and T3, the CMNPs group elicited a statistically significant mean increase compared to the control group, *p* < 0.001 (Table 2). 

### 3.4. Dynamic Evolution of the Body Weight as a Possible Tool to Evaluate the Efficiency of CMNPs Treatment 

**Figure 9:**
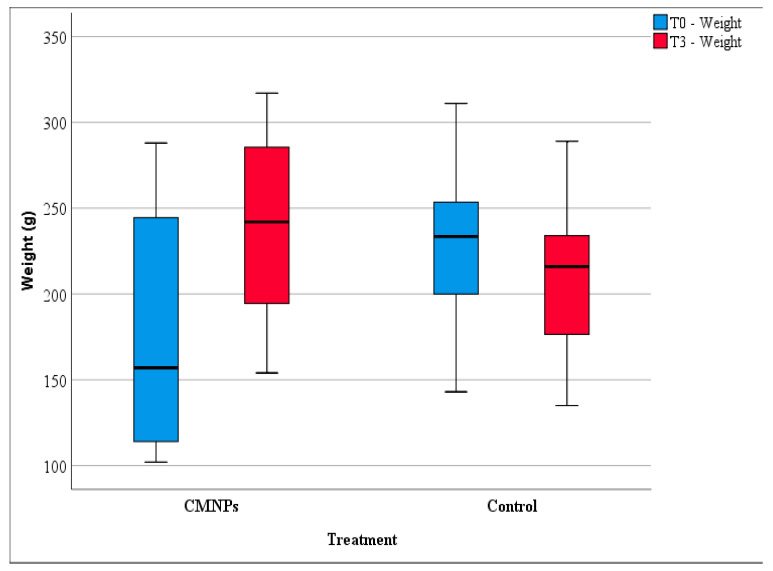
Comparative analysis of animal body weight evolution for CMNPs group and control group.

The animals from both groups were weighed before the peripheral nerve injury and at the end of the experiment, on day 21. The statistical analysis is rendered in Table 3 and Figure 9. Based on this analysis, the animals from the control group encountered a constant weight loss during the experiment, whereas the rats from the experiment group gained weight. These results, the weight gain observed in the animals that received CMNPs could be interpreted based on a favorable clinical and overall evolution of this group, where the animals presented minimum pain level, which might be what determined them to maintain or even increase their appetite. On the other hand, the rats from the control group lost weight, results that appeared to make body weight a possible indicator of the poor general state of these animals due to their increased level of pain and poor functional rehabilitation, transposed into a decreased appetite. Comparing the weight for T0 and then for T3, the CMNPs group mean was not statistically significant compared to the control group mean, *p* > 0.05 (Table 3).

Comparing the weight scores for CMNPs treatment revealed that T3 mean was significantly higher than T1 mean (*p* < 0.01). Comparing the weight scores for control treatment revealed that T3 mean was significantly lower than T1 mean (*p* < 0.001) (Table 3).

### 3.5. CMNPs Treatment Efficiency—Histological, TEM and EDX Nerve Studies

At the end of the experiment, on day 21, after euthanasia, right sciatic nerve samples were taken from all the animals and analyzed for the presence of any signs of nerve regeneration. 

**Figure 10:**
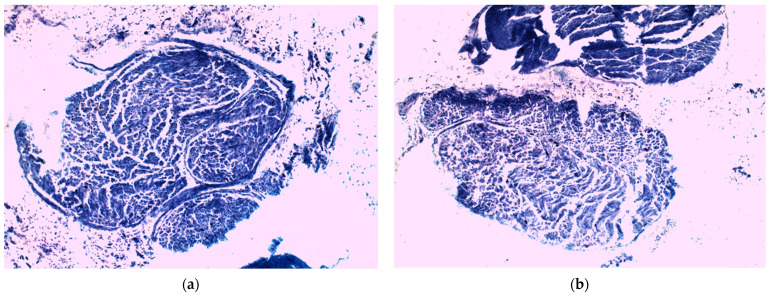
Right sciatic nerve samples—histological studies. (**a**) Control group nerve sample; (**b**) CMNPs treatment group nerve sample.

**Figure 11:**
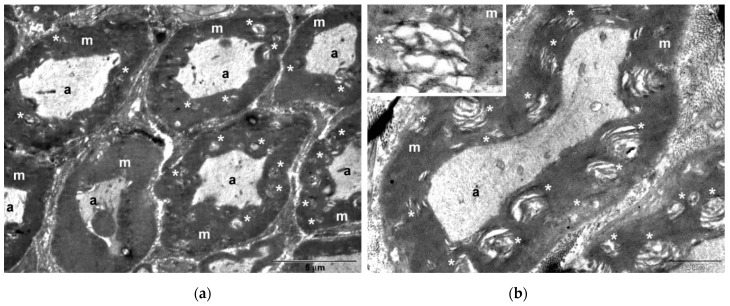

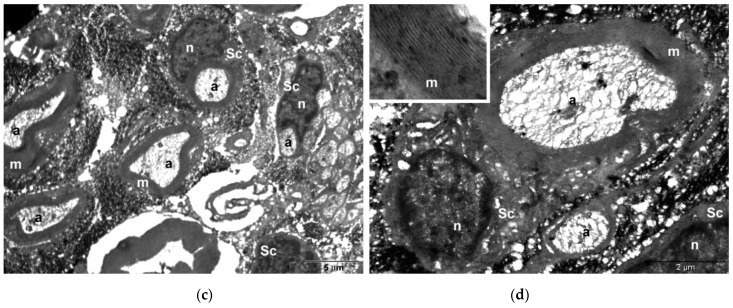
Right sciatic nerve samples—TEM studies. (**a**,**b**) Control group nerve sample; (**c**,**d**) CMNPs treatment group nerve sample. a, axon; m, myelin; n, nucleus; Sc, Schwann cell; *, region of disorganization of myelin layers.

In the analysis of the right sciatic nerve samples from animals in both groups, the same differences were observed, in histological and especially in TEM analysis. Regarding the right sciatic nerve of the animals in the control group, multiple myelin sheath disorganization sites were noticed (Figure 10a and Figure 11a,b), which are in fact disorganizations of the plasmalemma extensions of Schwann cells that surround the axons. The loss of axonal myelin layers’ continuity could be described as ovoid shaped areas of rarefaction (inset of Figure 11b), which induced a degeneration of the axon into debridements that followed the original endoneural tube. These changes in the compaction of the myelin sheath could be associated with a low expression of transmembrane myelin-specific proteins that are involved in its compaction. As a result, hypomyelination or demyelination occurred and, therefore, it resulted into a poor nerve recovery. These findings correlated with what the studied revealed about the evolution of the control group from the perspective of the SFI score, pain level, and body weight.

In comparison with the control group, the right sciatic nerve of the animals that received CMNPs treatment had almost normal morphology with discrete to moderate internal disorganization (Figure 10b and Figure 11c,d). The frequency of the ovoid areas of myelin disorganization was suggestively lower than in the control group. Moreover, TEM images observed the nanoparticles mostly at the axon and myelin sheath level as small black oval shape dots.

**Figure 12:**
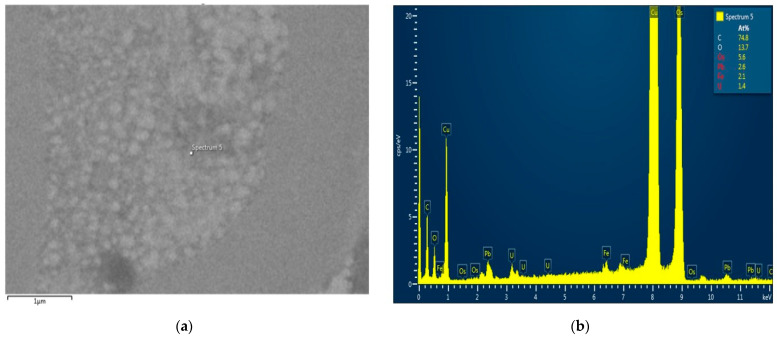
TEM-EDX analysis of right sciatic nerve sample from CMNPs group. (**a**) TEM image of the treated sciatic nerve; (**b**) Point-probe analysis using TEM equipped with EDX, showing the presence of iron (Fe) in the sciatic nerve.

The EDX analysis confirmed the presence of iron in the electron-dense accumulations identified inside the sciatic nerve samples of the treated group. The EDX data of the treated sciatic nerve, respectively the presence of iron shows that the iron oxide nanoparticles functionalized with chitosan were able to reach the sciatic nerve structure (Figure 12a,b).

### 3.6. CMNPs Treatment Efficiency—NGF Serum Values Dynamic Analysis

**Figure 13:**
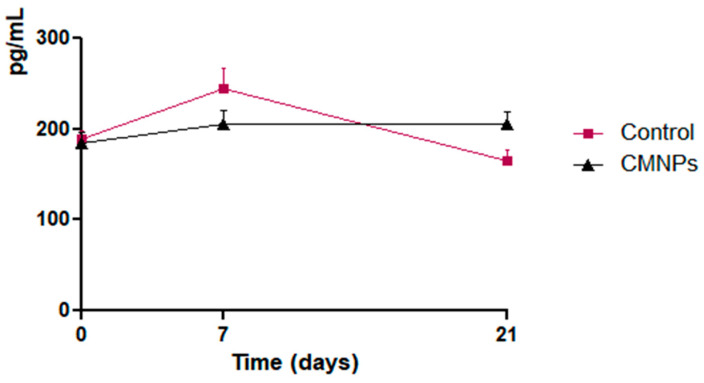
Comparative analysis of serum NGF values’ evolution for CMNPs group and control group.

There were no statistically significant differences between the two groups regarding the NGF serum levels, measured prior to the nerve lesion inducement, at seven days and 21 days after nerve injury (Figure 13). However, the group treated with CMNPs appears to have slightly constant increasing values of NGF serum levels when analyzing the levels from the beginning of the experiment until day 21. By comparison, the animals from the control group presented an elevated concentration of NGF at seven days after the sciatic nerve injury, but after this point, the NGF values followed a constantly descending path until day 21. 

## 4. Discussion

The results of the study demonstrated that the animals that received CMNPs oral treatment daily for 21 consecutive days obtained a statistically significant optimal functional rehabilitation, a decreased response to pain-like behavior evaluation, and a gain of total body weight compared to the control group. Although the serum NGF levels were not statistically significant between the groups, the NGF levels in the treated group indicated that CMNPs can stimulate the release of NGF and may contribute to peripheral nerve regeneration. These findings were confirmed by the histological and TEM images and encourage the study hypothesis that the daily oral administration of CMNPs could be a treatment capable to generate a good, even an excellent, peripheral nerve rehabilitation, according to Medinaceli criteria. 

Overall, the animals treated with CMNPs had a good functional recovery after peripheral nerve injury, whereas the control group presented poor functional rehabilitation or motor deficit at the end of the experiment. These results can be correlated with literature data, in which the beneficial effects of chitosan and iron oxide nanoparticles (in different other combinations than with chitosan) have been demonstrated.

Regarding iron oxide magnetic nanoparticles, different in vitro studies have shown that iron oxide magnetic nanoparticles prolong the activity of growth factors, like NGF, GDNF (glial derived nerve factor), and basic fibroblast growth factor (FGF-2) with important roles in nerve regenerative process [48,49]. Another advantage of MNPs is that they can be guided by magnetic fields, and in this way, the functionalized particles can be directed to the center of the nerve conduit where the concentration of secreted factors and cell localization is usually minimal during the normal physiological regeneration process [49]. However, the present study wanted to observe if the passive targeting mechanism, through which the nanoparticles are able to target specific sites mentioned by the literature in oncology therapy can be a possible mechanism also for peripheral nerve injuries, respectively if, without the intervention of an exterior magnetic field, the nanoparticles can be absorbed at the injury site. Another purpose was to see if CMNPs can reach and produce any effects at the nerve lesion site by oral administration. In this way, we appreciated that this type of non-invasive treatment could become a good option in cases of small, non-complete peripheral nerve injuries.

As literature data suggests, chitosan tubes proved to efficiently bridge peripheral nerve defects, but moreover, chitosan administrated orally seems to have similar outcome in non-complete nerve defects, like in the present research. Therefore, in peripheral nerve injuries that do not require surgery or when surgery is not possible, chitosan administrated orally, daily, appears to achieve a good to excellent functional recovery, as Xu et al. [50] and Ao et al. [51] presented in their studies, data that are concordant with our findings. The fact that chitosan has benefic effects on peripheral nerve injury recovery not only when administrated locally, but also when given systemically represents an important aspect to be considered in the future of treating peripheral nerve injuries. 

In our study, the animals that received CMNPs treatment gained weight (significantly different from the control group), which can be interpreted as a sign of a good general health with increased appetite and low or no distress. Moreover, the results are consistent with literature data, for example Meyer et al. measured the weight of tibialis anterior and gastrocnemius muscles taken from the ipsilateral injured side in animals treated with chitosan nerve guides, which was higher than the weight of the muscles in the control group [52]. Although most studies compared the muscle weight of the injured limb with the muscle weight of the healthy limb, we consider that the measurement of the total body weight stands as an interesting assessment tool of the animal’s general behavior and health. We believe that one of the causes of the body weight loss of the control group can be due to the atrophy of the muscles innervated by the affected nerve or to the motor impairment that caused the animals moving difficulties and, therefore, a possible poor food reach. Another potential cause could be the intensified pain-like behavior observed at the control group, which can be associated with a decreased appetite. 

The study’s findings that CMNPs treatment seems to reduce the pain level of the animals is an important aspect considering that most peripheral nerve injury patients confront with medium to severe pain, a major cause of disability [53]. Moreover, the presence of pain and other peripheral nerve injury symptoms can cause psychological problems, manifested through insomnia, anxiety, depression and overall, a decreased quality of life, which can lead to the emergence of chronic cortical pain and the reduction of brain plasticity of the hippocampus [54]. The consistent presence of pain that was observed at the animals in the control group could also partially explain the decreased appetite and loss of body weight. The statistically significant differences between the two groups regarding body weight, we interpreted to be correlated to the results obtained for the other studied parameters and to be an overall evaluation tool of the general state of the animals.

In histological and TEM analysis, the right sciatic nerve from the CMNPs group had more compact myelin sheath and a more structural organized axon, which could be translated into a superior functional outcome and could explain the higher SFI score, the better pain resistance, the minimal or no motor impairment and the increased appetite observed during those dynamic measurements. Moreover, TEM images captured the presence of the nanoparticles at the level of the axon and also in the myelin sheath, similar with the findings of other studies [49]. Plus, from our point of view, there is a significant difference between the two groups’ TEM images, in which the control group sciatic nerve had a more disorganized structure, especially at the level of the myelin sheath, compared to the CMNPs group. The EDX analysis demonstrated the presence of iron in the nerve samples of the CMNPs group. Our results transposed into the fact that the CMNPs were able to reach the injury site without any exterior intervention. This implies that oral administrated magnetic nanoparticles could represent a future treatment perspective like any other oral dispensed drug. Regarding MNPs used in peripheral nerve injuries, literature data showed that TEM nerve images, one week after median nerve injury induced in rats, revealed that some MNPs were localized within primary endosomes, endolysosomes and heterolysosomes and other MNPs were found in the cell cytoplasm [52]. In the same study, TEM images showed macrophages digesting degenerated myelin, part of the nerve regeneration process. The study also revealed that MNPs were present near the area of regenerating axons even at three weeks after injury [52]. The literature data are consistent with our study findings regarding the nanoparticles capability to remain in the nervous structures for a long period of time, which is an important aspect in the long-term treatment of peripheral nerve injuries. 

Other studies researched the effects of different types of nerve conduits as vehicle for the delivery of GFs such as NGF (nerve growth factor) for peripheral nerve treatment or the ability of NGF administrated orally to enhance peripheral nerve regeneration [8,10,55]. These studies used NGF as a therapeutic agent for the stimulation of peripheral nerve regeneration and have demonstrated that NGF substantially improved axonal regeneration, had neurotropism and neurogenesis effects and significantly reduced the regrowth and re-myelination time by stimulating myelin debris clearance, augmenting the number and diameter of myelinated nerve fibers [8]. By correspondence to other studies’ findings, in which serum NGF levels were severely decreased in diabetic polyneuropathy patients compared to healthy patients, we appreciate that our study results are consistent with literature data and suggest that the increased NGF serum levels observed in the CMNPs group can be correlated to a better peripheral nerve regeneration, confirmed by the other parameters analyzed [56]. Moreover, by using iron oxide magnetic nanoparticles in the present research, we appreciate that these particles had a role in stimulating the secretion of NGF, which promotes peripheral nerve regeneration as mentioned above. It would be interesting to observe if the NGF serum levels continue to increase for a longer period of time than the 21 days looked upon in our study, which could be a subject to be considered for future research.

Regarding the mechanism through which the CMNPs reached the nerve injury site, more studies are needed as we can only assume that there was a passive targeting process involved. Referring to IONPs administrated orally, most studies focused on toxicity analysis and very few on the mechanism of absorption and delivery through the gastrointestinal tract [57,58]. Our study, in contrast with the Chamorro et al. results [59], demonstrated the presence of CMNPs in the nerve at the lesion site, pointing out that the oral administration may be used as an effective treatment route that permits the accumulation of these nanoparticles not only in splanchnic organs after intraperitoneal or intravenous administration as previous researchers showed [60], but also in the nervous system.

According to the studied parameters, the dynamic evolution of the group that received CMNPs, analyzed alone or in comparison with the control group, presented significantly improved functional nerve rehabilitation, therefore, the hypothesis from which the study developed was confirmed. To the best of our knowledge, magnetic nanoparticles functionalized with chitosan administrated daily in a soluble form as a treatment method for peripheral nerve injuries have not been studied before. On the other hand, regarding the use of magnetic nanoparticles as viable transport systems for the delivery of various studied substances and the use of chitosan as a treatment method in different pathologies, the reported findings in this study are similar with those in literature. Therefore, the present study results suggest that CMNPs have the potential to enhance functional rehabilitation and pain reduction after peripheral nerve injury.

Besides the encouraging findings, our study presents some limitations. From one point of view, it can be considered that the number of animals used for the experiment is relatively small and cannot conduct to a strong statistical conclusion. This apparently low number of animals was chosen in consideration for the ethical principles of animal experiments, but, even in these conditions, the obtained statistical results were significant. It is appreciated that, for future research, the design of the study might be modified and include a larger number of animals, for a better assessment of the results.

From another point of view, it can be argued that the measurements of the SFI and pain-like behavior assessment are partially subjective methods, not very precise, although they are standardized and used for many years now. This precise deficiency is present despite of the measurements being done by two evaluators. On the other hand, all rodent studies regarding peripheral nerve injuries continuously used the SFI measurement method since it was developed in 1982 by Medinaceli et al. and modified in 1989 by Bain et al. to evaluate the functional rehabilitation level after a peripheral nerve injury [38]. Since then, no other method has been developed or considered sufficiently appropriate in these types of studies. Pain-like behavior evaluation in rodents like the Randall–Selitto test used in the present research is an assessment utilized in many in vivo studies and, by correlation with other evaluated parameters, represents a useful appraising method of the animal’s overall health state. In these conditions, finding new methods or improving the old ones for the SFI and pain-like behavior measurements would be welcome, though hard to obtain at this point in time. 

For future studies, it would be interesting to observe if the presence of MNPs in combination with chitosan may enhance chitosan’s favorable effects on peripheral nerve injury regeneration by comparing the results with a group that would receive simple chitosan solution. Future studies could also investigate the effects of the CMNPs for a longer period of time than 21 days, to see if the MNPs remain present in the nerve structures and to evaluate the possible side effects and toxicity of these particles. 

## 5. Conclusions

CMNPs orally administrated treatment had beneficial effects on the rehabilitation of peripheral nerve injuries regarding all studied parameters: SFI score, pain-like behavior measurement, body weight dynamic evolution, serum NGF levels, and histological-ultrastructural studies. By comparing these parameters and statistically analyzing the results, the differences between the control group and the group that received daily treatment with CMNPs were significant. Moreover, EDX analysis demonstrated that CMNPs are able to reach the sciatic nerve structure without any exterior intervention. Even though the study findings are promising and represent a step forward in the developing of a conservative treatment capable of generating optimal peripheral nerve regeneration, more studies are needed in order to formulate a more accurate conclusion and to make possible a transition of the research to the next phase of human testing.

## Figures and Tables

**Table 1:** SFI score after Medinaceli’s formula (1982).

SFI Score	Functional Rehabilitation Degree
12 to −12	Excellent
−13 to −37	Good
−38 to −62	Medium
−63 to −87	Non-satisfactory
−88 to −137	Complete deficit

**Table 2:** Comparison of mean pain scores between the two treatment groups for the experimental foot.

		CMNPs Treatment		Control Treatment		Independent *t*-Test
		Mean	SD	Mean	SD	*p*-Value
**Experimental foot pain level**	T0	192.50	18.90	195.63	16.78	0.732
	T1	194.38	13.74	144.38	14.00	<0.001
	T2	186.25	13.30	107.50	15.58	<0.001
	T3	190.63	12.66	81.25	9.16	<0.001

**Table 3:** Comparison of mean body weight measurements between the two groups for the experimental foot.

		CMNPs Treatment		Control Group		Independent *t*-Test
		Mean	SD	Mean	SD	*p*-Value
Weight (g)	T0	177.63	72.04	228.5	50.56	0.124
	T3	239.37	58.06	209.62	49.03	0.287

## Data Availability

Not applicable.
